# Lamellar macular defects: are degenerative lamellar macular holes truly degenerative?

**DOI:** 10.3389/fmed.2023.1156410

**Published:** 2023-04-17

**Authors:** Grazia Pertile, Daniela Iacovello, Giorgia Maraone, Elisa Bottega, Massimo Guerriero, Emilia Maggio

**Affiliations:** ^1^Department of Ophthalmology, IRCCS Sacro Cuore Don Calabria Hospital, Verona, Italy; ^2^Clinical Research Unit, IRCCS Sacro Cuore Don Calabria Hospital, Verona, Italy

**Keywords:** macular defect, healing process, vitrectomy, lamellar macular hole, ERM foveoschisis

## Abstract

**Purpose:**

To investigate morpho-functional changes after surgical treatment for ERM foveoschisis or lamellar macular hole (LMH), and to evaluate whether the two entities are associated with different healing processes and long-term outcomes.

**Design:**

Retrospective interventional case series.

**Methods:**

A total of 56 eyes, treated for lamellar macular defects and followed up for 24 months, were enrolled. The eyes were divided into two groups: 34 with ERM foveoschisis and 22 with LMH. Changes in the following features were evaluated and compared between the two groups: best-corrected visual acuity (BCVA), external limiting membrane (ELM) and ellipsoid zone (EZ) defects, central foveal thickness (CFT), and autofluorescence (FAF) diameter and area.

**Results:**

After surgery, progressive BCVA improvement was observed with no significant difference between the two groups (*p*-value: 0.06). An increased number of eyes with intact outer-retinal layers was found both in the ERM foveoschisis and LMH groups. FAF diameter and area decreased significantly throughout the FU with no significant difference between the two groups (*p*-value: 0.2).

**Conclusion:**

In the present study, significant functional and microstructural improvements were observed after surgery for both ERM foveoschisis and LMH, demonstrating considerable repair potential in both types of lamellar defects. These findings question the true “degenerative” nature of LMH.

## 1. Introduction

The improved resolution of the latest generation of OCT has significantly impacted the understanding of non-full thickness macular holes. Witkin et al. (1) used ultra-high definition OCT to set the criteria for diagnosing lamellar macular holes (LMH). Subsequently, the introduction of spectral domain OCT (SD-OCT) enabled clinicians to identify peculiar characteristics for both the epiretinal tissue (2–4) and the foveal microstructure (5, 6). These insights suggested the existence of distinct entities with a different pathogenesis and evolutionary path. Some lamellar defect-associated epiretinal membranes (ERM) may have primarily contractile properties (3), and thus, in these cases, the pathogenesis is likely related to a tractional stimulus. On the other hand, a different type of epiretinal proliferation has been defined as “thick membrane” (2) or lamellar hole-associated epiretinal proliferation (LHEP) (4), based on specific OCT and immunohistochemical characteristics (2, 3, 7). Different studies have reported that LHEP is often associated with ellipsoid zone (EZ) and external limiting membrane (ELM) defects, as well as worse visual acuity at baseline (8–11). Based on these findings, Govetto et al. (6) described this kind of lamellar foveal defect as “degenerative.” Consequently, many authors have adopted this term to describe LMH.

Recently, a panel of experts reached a consensus on the definition of non-full thickness macular defects and their distinguishing features (12). Three entities were identified based on OCT characteristics, namely: LMH, epiretinal membrane (ERM) foveoschisis, and macular pseudohole (MPH). This classification, that is used throughout the present study, may allow a greater concordance in non-full thickness macular defect definition in future studies, making findings more comparable and thus helping toward the achievement of definitive conclusions.

Although several previous studies have examined non-full thickness macular defects and their evolution and changes after treatment, there is still a lack of conclusive evidence. Both ERM foveoschisis and LMH have been considered rather benign disorders, as in many cases the disease remains stable or advances very slowly over time. However, studies with longer follow up periods have demonstrated the progression of these diseases, suggesting a slow, possibly degenerative evolution (13). Moreover, some authors have reported no visual improvement in LMH after surgery (8, 14, 15), which supports the degenerative hypothesis. However, there is increasing evidence that surgery may also be beneficial for patients with LMH (9–16). This raises speculation regarding the true degenerative nature of LMH, since it remains to be understood whether a condition that can improve after surgery, and in most cases does not recur, can be defined as “degenerative.”

Therefore, the purpose of the present study was to investigate the microstructural and functional evolution of a series of eyes that underwent surgical treatment for ERM foveoschisis or LMH, to evaluate whether the two entities are associated with a different healing process, and to examine the influence of the healing process on long-term outcomes.

## 2. Materials and methods

We conducted a retrospective interventional case-series study including consecutive eyes that underwent surgery for non-full thickness macular holes at the Sacro Cuore Hospital between January 2008 to January 2018.

As a routine practice at the Sacro Cuore Hospital, a surgical approach was considered for patients that lost two or more lines of BCVA during the follow up and showed a documented progressive deterioration in outer retinal layers, and for eyes that had a BCVA at presentation of less than 0.3 logMAR and complained of significant metamorphopsia. Supplementary Figure 1 shows an example of a case followed-up and then treated with surgery.

Only patients with a minimum follow up (FU) of 24 months were included, and all analyses were performed over this period. Exclusion criteria were prior retinal surgery, the presence of choroidal neovascularization, diabetic retinopathy, retinal vascular diseases, infectious diseases, uveitis, high myopia, or other conditions that could influence best corrected visual acuity (BCVA), except for lens opacity.

The present research adhered to the tenets of the Declaration of Helsinki. Informed consent was obtained from all the patients, and the study was approved by the Sacro Cuore Hospital Institutional Review Board.

### 2.1. Evaluation procedures

At baseline and at each post-operative visit (1, 3, 6, 12, and 24 months after surgery), all eyes underwent a complete ophthalmologic examination, including BCVA assessment with Snellen visual charts, measurement of intraocular pressure, biomicroscopic examination, dilated fundus examination with a 90-diopter indirect lens, SD-OCT examination, and blue-fundus autofluorescence (FAF). The BCVA was converted to logMAR equivalents for statistical analysis.

OCT was performed with SD OCT-SLO (Heidelberg Engineering, Heidelberg, Germany). The same OCT was used through the study period. The routine scanning protocol at the Sacro Cuore Hospital consisted of both an 8 mm crosshair scan (with two sections perpendicular to each other) and a posterior pole series consisting of 128 horizontal B-scan images. Each image was composed of 512 axial scans, covering an 88 mm^2^ area of the posterior pole. Baseline scans were set as references for the ensuing FU examinations, using the automatic software provided by the instrument.

Fundus autofluorescence images (excitation 488 nm, emission >500 nm) were recorded with a confocal scanning laser ophthalmoscope (Heidelberg Retina Angiograph, HRA classic; Heidelberg Engineering, Dossenheim, Germany).

### 2.2. SD-OCT classification of lamellar macular defects

As mentioned, we used the new criteria from the international panel of vitreoretinal experts to classify the lamellar macular defects. The eyes were thus divided into two groups, as follows:

• Group 1, ERM foveoschisis: presence of contractile ERM and schisis at the level of Henle’s fiber layer. Optional anatomical features: presence of microcystoid spaces in the inner nuclear layer (INL), an increase of retinal thickness, and presence of retinal wrinkling.
• Group 2, LMH: presence of irregular foveal contour, foveal cavity with undermined edges, and at least one other sign of foveal tissue loss (pseudo-operculum, thinning of the foveal at its center or around). Optional anatomical features: presence of LHEP, a central foveal bump, and EZ interruption.

Contractile ERM was defined as a thin, hyper reflective line on the epiretinal surface, LHEP as a thick, homogeneous, and isoreflective material in close contact with the internal limiting membrane (ILM).

### 2.3. Evaluation of SD-OCT morphologic features

At each time point, the following SD-OCT features were evaluated and compared between the two groups:

• ELM and EZ integrity, evaluated through horizontal and vertical scans and defined as intact when the line was continuous, and disrupted when interrupted or absent.
• ELM and EZ defect size, evaluated through horizontal and vertical scans with the built-in manual spectral caliper.
• central foveal thickness (CFT), measured with the built-in manual spectral caliper function, and defined as the smallest vertical distance from the base of the lamellar defect to the hyper reflective retinal pigment epithelium layer.

Correlations between these SD-OCT morphologic parameters and BCVA were analyzed.

### 2.4. Autofluorescence analysis

Major horizontal and vertical diameters, as well as the total FAF area, were measured with the built-in manual spectral caliper function and compared between the two groups.

### 2.5. Surgical procedure

All patients underwent 23G pars plana vitrectomy. The ERM was removed, and the ILM was stained with brilliant blue G and trypan blue (Membrane Dual; Dorc, Zuidland, The Netherlands) for approximately 30 s, and then peeled off in a circular fashion approximately three disk diameters around the LMH. Air or 14% SF6 gas tamponade were used. Particular attention was paid while removing the LHEP from the fovea. The LHEP was cut with scissors whenever the tissue could not be easily removed from the edge of the LMH with forceps.

Vitrectomy was combined with phacoemulsification in eyes with cataract. The number of eyes underwent vitrectomy combined with phacoemulsification was evaluated. Moreover, the number of eyes underwent phacoemulsification in the follow up period was also assessed.

### 2.6. Statistical analysis

Demographic and clinical data were summarized using descriptive statistics, measures of variability, and precision. Statistical tests were used based on the type of variables, test assumptions and sample dimension. In detail, a K-S test was used to test the normality in distribution for continuous variables. The ordinal two sample *t*-test or the non-parametric Wilcoxon rank-sum test was used to compare the mean of two independent groups of units (Group 1 and Group 2).

Paired *t*-test or the non-parametric Wilcoxon matched-pairs signed-rank test was performed to compare the mean of dependent groups.

The Pearson coefficient was used to estimate the linear correlation between normal distributed continuous variables.

A *p*-value less than 5% was considered as being statistically significant.

The analysis were performed by STATA (StataCorp., 2019, Release 16. College Station, TX, USA).

## 3. Results

A total of 56 eyes met the inclusion criteria and were enrolled in the study. Baseline characteristics of the study population are reported in Table 1. Group 1 (ERM foveoschisis) included 34 eyes (60.7%), while group 2 (LMH) included 22 eyes (39.3%). Five eyes of group 2 exhibited both contractile ERM and LHEP. Although baseline BCVA was slightly lower in the LMH eyes, no statistically significant difference was detected between the two groups (*p*-value: 0.14).

**TABLE 1:** Demographics and clinical characteristics of study population, expressed in terms of mean, standard deviation, and percentage.

	Group 1 ERM foveoschisis (*N* = 34)	Group 2 lamellar macular hole (LMH) (*N* = 22)
Age in years mean (SD), min-max	70.2 (8.81), 55–85	73.1 (8.63), 55–87
Sex *N* male/female	9/25	17/5
Preoperative pseudophakia (eyes) *N* (%)	4 (11.7)	7 (31.8)
Combined cataract extraction (eyes) *N* (%)	24 (70.5)	12 (54.5)
Preoperative BCVA (Logmar) mean (SD)	0.28 (0.15)	0.33 (0.16)
Final BCVA (Logmar) mean (SD)	0.07 (0.10)	0.18 (0.24)
Preoperative AF area (mm^2^) mean (SD)	0.23 (0.18)	0.23 (0.13)
Final AF area (mm^2^) mean (SD)	0.05 (0.11)	0.05 (0.10)
Preoperative AF horizontal diameter (μm) mean (SD)	348.04 (167.52)	409.93 (159.17)
preoperative AF vertical diameter (μm) mean (SD)	324 (159.70)	371.23 (167.18)
Preoperative CFT (μm) mean (SD)	182.38 (50.23)	122.95 (45.01)
Final CFT (μm) mean (SD)	287.85 (97.57)	178.14 (52.89)
Intact preoperative ELM (eyes) *N* (%)	32 (94.11)	4 (18.18)
Intact preoperative EZ (eyes) *N* (%)	32 (94.11)	18 (81.81)

Vitrectomy was combined with phacoemulsification in 36 eyes (24 eyes in group 1, 12 eyes in group 2). The number of eyes that underwent phacoemulsification in the follow up period is reported in Supplementary Table 1. The range of preoperative refractions in eyes that underwent phacoemulsification is reported in Supplementary Table 2.

After surgery, a progressive and continuous improvement in BCVA was recorded, both in patients with ERM foveoschisis and in those with LMH (Table 2). BCVA improvement became statistically significant earlier in eyes with ERM foveoschisis (1 month post-op; *p*-value: 0.004) compared to LMH (6 months post-op; *p*-value < 0.0001). However, there was no significant difference in final visual improvement between the two groups (*p*-value: 0.09).

**TABLE 2:** BCVA (Logmar) analysis over 24 months follow up time.

	Baseline (t_0_)	1 month (t_1_)	3 months (t_2_)	6 months (t_3_)	12 months (t_4_)	24 months (t_5_)
ERM foveoschisis mean (SD)	0.28 (0.15)	0.18* (0.15)	0.17 (0.15)	0.13 (0.13)	0.10 (0.12)	0.07 (0.10)
LMH mean (SD)	0.33 (0.16)	0.33 (0.29)	0.26 (0.25)	0.22^†^ (0.24)	0.22 (0.25)	0.18 (0.24)

*Statistically significant improvement in BCVA compared to baseline (*p*-value: 0.004).

^†^Statistically significant improvement in BCVA compared to baseline (*p*-value < 0.0001).

Multivariate linear regression model was performed to analyze influence of cataract surgery on visual improvement. We found no statistically significant influence adjusted for baseline BCVA (*p* = 0.122).

### 3.1. Evaluation of SD-OCT morphologic features

At baseline, ELM and EZ defects were mostly present in LMH eyes. Only two eyes with ERM foveoschisis presented defects in both external bands, while 18 (81.8%) and 4 (18.2%) of LMH eyes exhibited disrupted ELM and/or EZ, respectively.

The OCT-scan analysis showed a progressive increase in the number of eyes with intact outer-retinal layers throughout the FU. At the end of the observation period, intact EZ and ELM were recorded in all ERM foveoschisis eyes, while only two eyes in group 2 showed a persistent disruption in both EZ and ELM, with five eyes showing a disruption in the EZ alone.

**FIGURE 1:**
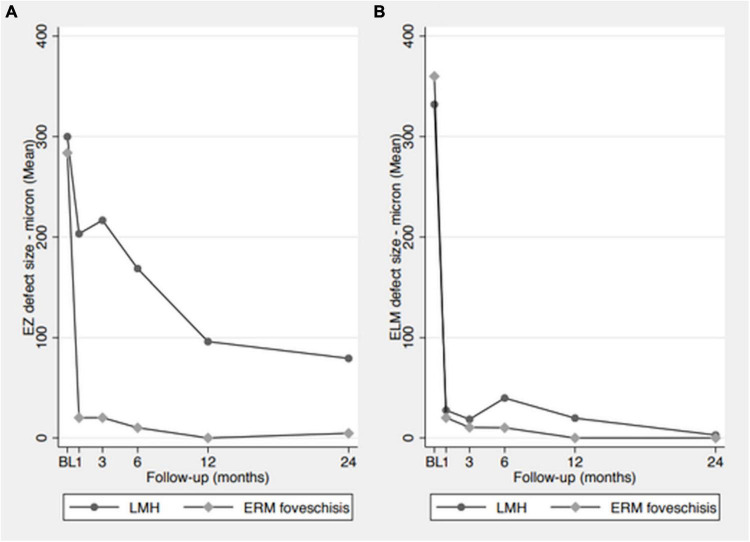
Analysis of ELM and EZ defect size in both groups at each time point. **(A)** EZ defect size measurements, showing a progressive size reduction in LMH group and a rapid reduction in ERM foveoschisis group. **(B)** ELM defect size measurements, showing a rapid reduction in the defect size in both groups.

Analyses of ELM and EZ defect size in both groups at each time point are shown in Figure 1.

**FIGURE 2:**
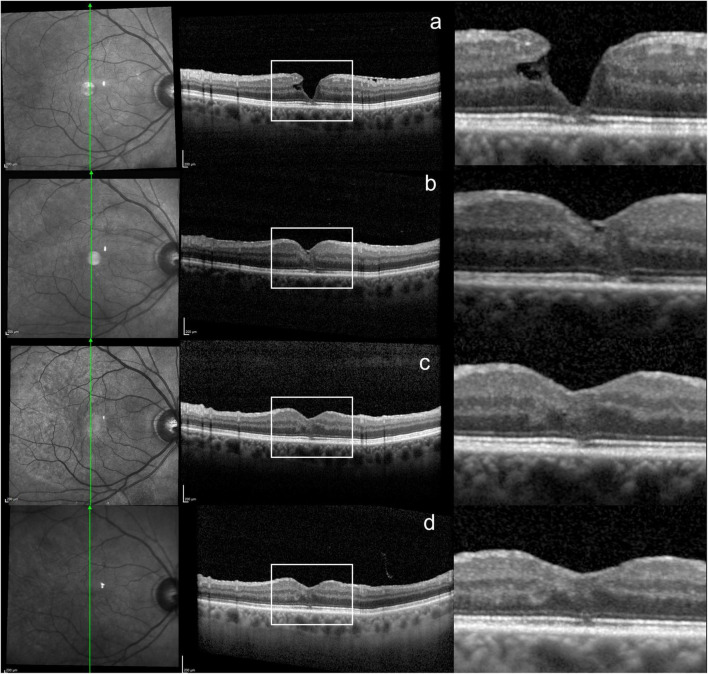
Progressive healing of the outer retinal layers over a 2-year period in a LMH eye. **(a)** SD-OCT scan at baseline, showing disrupted ELM/EZ. **(b,c)** 3 and 6 months after surgery, showing a reduction in the ELM/EZ defect. **(d)** Last visit, 2 years after surgery, showing further ELM/EZ restoration.

The healing process of the ELM and EZ was particularly slow in some LMH eyes and continued during the second year of FU. Figure 2 shows an example of the progressive recovery of the outer retinal layers over a 2-year period. An additional example is shown in Supplementary Figure 2. None of the eyes with LHEP developed a LHEP recurrence throughout the FU.

At baseline, a statistically significant correlation was found between BCVA and outer-retinal layer integrity, since BCVA was significantly lower in eyes with external band defects (*p*-value: 0.03). The correlation between BCVA and outer layer integrity was also evaluated at the successive time points, showing a significant correlation. However, considering the small number of eyes with EZ/ELM defects in the last visits, the analysis holds little statistical significance.

Central foveal thickness measurements are reported in Table 3. No statistically significant differences were found between the two groups either at baseline or at each successive time point. CFT increased significantly in both groups at 1 month (*p*-value < 0.0001) and decreased slightly at the successive timepoints (Supplementary Figure 3).

**TABLE 3:** CFT (μm) in the two groups at baseline and at each time point.

	Baseline (t_0_)		1 month (t_1_)		3 months (t_2_)		6 months (t_3_)		12 months (t_4_)		24 months (t_5_)	
ERM foveoschisis mean (SD)	182.38 (50.23)	*p*-value: 1	330.35 (108.40)	*p*-value: 0.99	325.31 (99.41)	*p*-value: 1	310.46 (90.74)	*p*-value: 1	302.76 (103.12)	*p*-value: 1	287.85 (97.57)	*p*-value: 1
LMH mean (SD)	122.95 (45.01)		217.94 (96.66)		209.57 (68.69)		204.28 (66.12)		194.09 (68.22)		178.14 (52.89)	

At baseline, a weak direct correlation was found between BCVA and CFT in LMH eyes (r 0.41), while in ERM foveoschisis eyes, no correlation was detected.

### 3.2. Autofluorescence analysis

**FIGURE 3:**
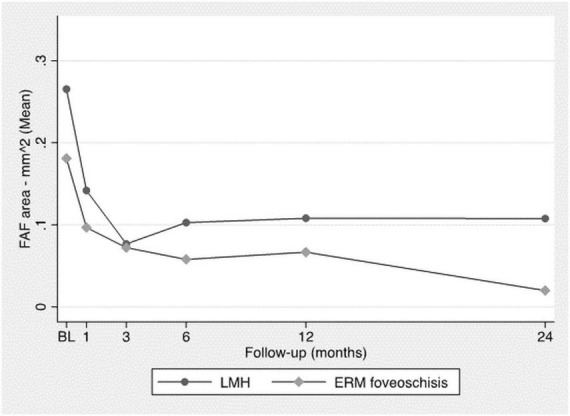
Analysis of FAF area through the FU.

Horizontal and vertical major diameters and the total FAF area decreased significantly throughout the FU (Figure 3) with no significant difference between the two groups (*p*-value: 0.2).

Supplementary Figure 4 shows an example of improvement in autofluorescence.

## 4. Discussion

A lamellar macular defect implies alterations of the inner retinal layers that may be associated with outer retinal changes and visual impairment. Its pathogenesis is hypothesized to be mainly tractional or degenerative, leading to differentiation between ERM foveoschisis and LMH. Based on this distinction, one may expect the evolution of these two entities to be diverse. In this study, we evaluated microstructural changes induced by surgery for different types of lamellar macular defects to better understand their origins and the possibly degenerative nature of LMH.

Some previous studies have reported that LMH appears to be associated with worse outcomes after surgery (14, 15), supporting the degenerative hypothesis, while others have found surgery to be beneficial for eyes showing disease progression (17, 18). However, there are many discrepancies between these studies, preventing definitive conclusions.

In agreement with other authors (10, 16, 19), we found a significantly higher incidence of outer retinal layer defects in LMH eyes at baseline compared to ERM foveoschisis. Nevertheless, at the end of the FU, 95% of the LMH eyes showed a complete recovery of the ELM and 77% of the EZ. In addition, a significant improvement in BCVA was observed in the whole population, with no statistically significant difference between the two groups. These findings suggest considerable potential for repair in both types of lamellar defects.

In contrast with the studies positing LMH as degenerative, we observed significant healing potential in eyes with LMH. This definition of “degenerative” therefore becomes questionable. Degeneration is usually considered as “a change of tissue to a lower or less functionally active form.” In this meaning, both LMH and ERM foveoschisis could be considered degenerative as they show morphological alterations and a certain degree of visual function loss. Instead, if one defines degeneration as “progressive and irreversible tissue deterioration,” LMH is best considered a non-degenerative disease as successful surgical intervention may not only stop the process, but also lead to microstructure recovery in a significant number of eyes.

In our study, another finding in opposition of the degenerative LMH hypothesis comes from the OCT morphologic analysis. In fact, we found that the CFT increased significantly at 1 month, likely because of the edematous reaction to surgery, and then decreased slightly in both groups throughout the successive timepoints. This may be a sign that after hole closure, there is no further tissue proliferation in either type of lamellar defect, suggesting a stationary condition. Moreover, we observed that the LHEP did not tend to recur after hole closure as one might expect in a degenerative process. Pang et al. (20) postulated that the LHEP originates from within the inner retinal defect as the epiretinal material observed in LMH has medium reflectivity, and is identical to the reflectivity seen in the middle retinal layers. They speculate that Muller cells may be involved in the formation process of this proliferation. Other authors hypothesized that Muller cells are the primary impetus for LHEP, given Muller cells likely produce it to heal the retinal defect (4, 21). From this point of view, we may assume that the LHEP is secondary to foveal cavitation, which, in turn, is caused by traction at the inner retina layer level. The traction release and consequential repair of the intraretinal splitting obtained with ILM peeling appears to stop the epiretinal tissue proliferation, and thus explains the lack of recurrence after successful surgery. Following this logic, one may hypothesize that traction is at the root of both ERM foveoschisis and LMH.

An increased FAF signal has been previously described in LMH and is supposed to correlate with an interruption of the outer plexiform layer, where the macular pigment is normally denser and partially blocks the macular lutein-induced autofluorescence (10, 22). Therefore, in our population, the progressive improvement in FAF parameters may be considered a sign of improved retinal conditions at the level of the outer plexiform layer due to hole closure. This was the same between the two groups, confirming the potential for LMH recovery.

In our study, the healing process of ELM and EZ was found to be particularly slow in some LMH eyes and continued during the second year of FU. Consequently, previous studies with a shorter FU may have missed part of the capacity for improvement in some eyes, thereby underestimating the potential for recovery in LMH cases. This may also explain the worse outcome reported for this type of lamellar defect in certain studies. Another reason for the worse outcome reported in previous studies may be the inclusion of eyes at advanced disease stages with a low baseline BCVA. In these eyes, long-lasting disease may have permanently impaired the photoreceptors, thus limiting recovery potential. For example, Choi et al. (23) reported a worse functional outcome in the LMH group compared with our results, and a lack of statistically significant improvement after surgery despite the long FU. One can deduce that in this study, eyes with more advanced disease were included as the mean baseline BCVA was worse in the LMH group (0,501 LogMAR vs. 0,360 LogMAR), suggesting long-lasting disease. Moreover, Obata et al. (24) reported a series of eyes with a higher mean preoperative BCVA (0.33 LogMAR vs. 0.30 LogMAR for LMH group and ERM foveoschisis group, respectively) and found no significant difference in BCVA between the two groups after 12 months, similar to our study. These observations suggest that the timing of surgery is likely to be crucial, as advanced stages of the disease may be associated with irreversible outer retinal layer damage.

A further explanation for the discrepancies between previous studies stems from the diverse parameters to evaluate surgical outcomes, namely visual acuity or the restoration of the anatomical appearance of the inner or outer retina. For example, Figueroa et al. (16) described the healing process based on the appearance of the inner retinal layers. From this point of view, they observed more frequent delayed healing in ERM foveoschisis, as the hyporeflective spaces in the foveal region disappeared gradually during the FU as compared to the general trend seen in LMH.

A final explanation for the worse outcomes in older studies is the difficulty in recognizing the presence of LHEP in the pre-SD OCT era, which implies less accuracy in the surgical strategy. In fact, an increase in LMH closure rate may be associated with an awareness of LHEP characteristics. As previously described (2), this is a sticky, soft material that can be quite difficult to remove. If the LHEP is not removed first, it is almost impossible to remove the ILM as it does not stain with dyes. This may explain why suboptimal anatomical results have been frequently described in the past (25, 26) since this entity was not well recognized before the introduction of SD-OCT. In addition, the LHEP originates or at least reaches the intermediate retinal layers. An attempt to remove this tissue, especially if it is well adhered to the inside of the foveal cavitation, may damage the retinal structure and form an FTMH, a post-operative event observed by many authors (17). In this cohort of patients, none developed this complication, likely due to increased attention during LHEP removal from the fovea. In the present study, the LHEP was cut with scissors whenever the tissue could not be easily removed from the edge of the LMH with forceps.

The limitations of this study are its retrospective nature and sample size. Nevertheless, it carefully observed microstructural changes during a 2-year FU that offer insight into the pathogenesis of this disease. A larger cohort of patients is necessary to draw more meaningful conclusions.

In conclusion, the present study found significant functional and microstructural improvements after surgery for ERM foveoschisis and LMH, demonstrating considerable repair potential in both types of lamellar defects. We therefore suggest a reconsideration of the degenerative definition of LMH, since it may not reflect the possibility for improvement after treatment.

## Nomenclature

### Resource identification initiative

To take part in the Resource Identification Initiative, please use the corresponding catalog number and RRID in your current manuscript. For more information about the project and for steps on how to search for an RRID, please click here.

### Life science identifiers

Life Science Identifiers (LSIDs) for ZOOBANK registered names or nomenclatural acts should be listed in the manuscript before the keywords with the following format: urn:lsid: authority:<Namespace>:<ObjectID>[:<Version>]

## Data availability statement

The original contributions presented in this study are included in the article/Supplementary material, further inquiries can be directed to the corresponding author.

## Ethics statement

The studies involving human participants were reviewed and approved by the Ethics Committee for Clinical Trials (CESC) of the Provinces of Verona and Rovigo based at the AOUI of Verona. The patients/participants provided their written informed consent to participate in this study.

## Author contributions

GP contributed to the design of the work, drafted the work, and provided approval for publication agreed to be accountable for all aspects of the work in ensuring that questions related to the accuracy or integrity of any part of the work are appropriately investigated and resolved. DI contributed to the design of the work, acquisition and interpretation of data, revised it critically, and provided approval for publication of the content. GM contributed to the design of the work, acquisition of data, revised it critically for important intellectual content, and provided approval for publication of the content. EB contributed to the acquisition of data, revised it critically for important intellectual content, and provided approval for publication of the content. MG and EM contributed to the design of the work, analysis or interpretation of data for the work, revised it critically for important intellectual content, and provided approval for publication of the content agreed to be accountable for all aspects of the work in ensuring that questions related to the accuracy or integrity of any part of the work are appropriately investigated and resolved. All authors contributed to the article and approved the submitted version.
