# Supplementary material for: Lamellar macular defects: are degenerative lamellar macular holes truly degenerative?
Source: Front Med (Lausanne). 2023 Apr 17;10:1156410. doi: 10.3389/fmed.2023.1156410 (PMC10149835; doi:10.3389/fmed.2023.1156410)
Supplement: Supplementary file 1 [file Data_Sheet_1.pdf]

Supplementary Material

Lamellar macular defects: are degenerative lamellar macular holes truly degenerative?

Grazia Pertile[*], Daniela Iacovello, Giorgia Maraone, Elisa Bottega, Massimo Guerriero, Emilia Maggio

* Correspondence:
Grazia Pertile
grazia.pertile@sacrocuore.it

Supplementary Figures Captions

Figure 1S. An example of indications to treatment: a case followed-up for 10 years and then treated with surgery.

(a) Ten years before surgery. BCVA: 0 logMAR (20/20 Snellen fractions). In the square, a magnification of foveal area shows intact outer retinal layers.

(b) Five years before surgery. BCVA: 0.2 logMAR (20/32 Snellen fractions). In the square, a magnification of foveal area shows disrupted outer retinal layers.

(c) One year before surgery. BCVA: 0.4 logMAR (20/50 Snellen fractions). In the square, a magnification of foveal area shows increasing disruption in intact outer retinal layers.

(d) Two years after surgery. BCVA: 0 logMAR (20/20 Snellen fractions). In the square, a magnification of foveal area shows restoration outer retinal layers.

Figure 2S. Progressive healing of the outer retinal layers over a 2-year period in a LMH eye.

(a) SD-OCT scan at baseline, showing disrupted ELM/EZ. BCVA: 0.4 logMAR (20/50 Snellen fractions).

(b) 3 months after surgery, showing a reduction in the ELM/EZ defect. BCVA: 0.3 logMAR (20/40 Snellen fractions).

(c) 12 months after surgery, showing a further improvement in ELM/EZ defect. BCVA: 0.2 logMAR (20/32 Snellen fractions).

(d) Last visit, 2 years after surgery, showing further improvement in outer retinal layers. BCVA: 0.2 logMAR (20/32 Snellen fractions).

Figure 3S. Analysis of CFT in both groups through the FU period.

Figure 4S. Progressive improvement in autofluorescence over a 2-year period.

(a) AF at baseline, showing iperautofluorescence at the foveal area.
(b) (c) 1 and 24 months after surgery, showing regression of iperautoflurescence at the foveal area.
