# Supplementary material for: Lamellar macular defects: are degenerative lamellar macular holes truly degenerative?
Source: Front Med (Lausanne). 2023 Apr 17;10:1156410. doi: 10.3389/fmed.2023.1156410 (PMC10149835; doi:10.3389/fmed.2023.1156410)
Supplement: Supplementary file 2 [file Table_1.pdf]

Table 1S. Number of eyes that underwent phacoemulsification in the follow up period.

Phacoemulsification during follow up		
Whole population (Total N. 56)	Group 1 ERM foveoschisis (Total N. 34)	Group 2 LMH (Total N. 22)
7	4	3
