# Supplementary material for: Lamellar macular defects: are degenerative lamellar macular holes truly degenerative?
Source: Front Med (Lausanne). 2023 Apr 17;10:1156410. doi: 10.3389/fmed.2023.1156410 (PMC10149835; doi:10.3389/fmed.2023.1156410)
Supplement: Supplementary file 3 [file Table_2.pdf]

Table 2S. Range of preoperative refractions in eyes that underwent phacoemulsification (both combined with vitrectomy and during the follow up period).

	Vitrectomy combined with phacoemulsification		Phacoemulsification during follow up	
	Group 1<br>ERM foveoschisis	Group 2<br>LMH	Group 1<br>ERM foveoschisis	Group 2<br>LMH
Number of eyes	24	12	4	3
Range of refractions (Dioptres)	0 / +3	0 / +1	+1 / +3.75	-4 / +1.5
