# Supplementary figures and images for: Lamellar macular defects: are degenerative lamellar macular holes truly degenerative?
Source: Front Med (Lausanne). 2023 Apr 17;10:1156410. doi: 10.3389/fmed.2023.1156410 (PMC10149835; doi:10.3389/fmed.2023.1156410)

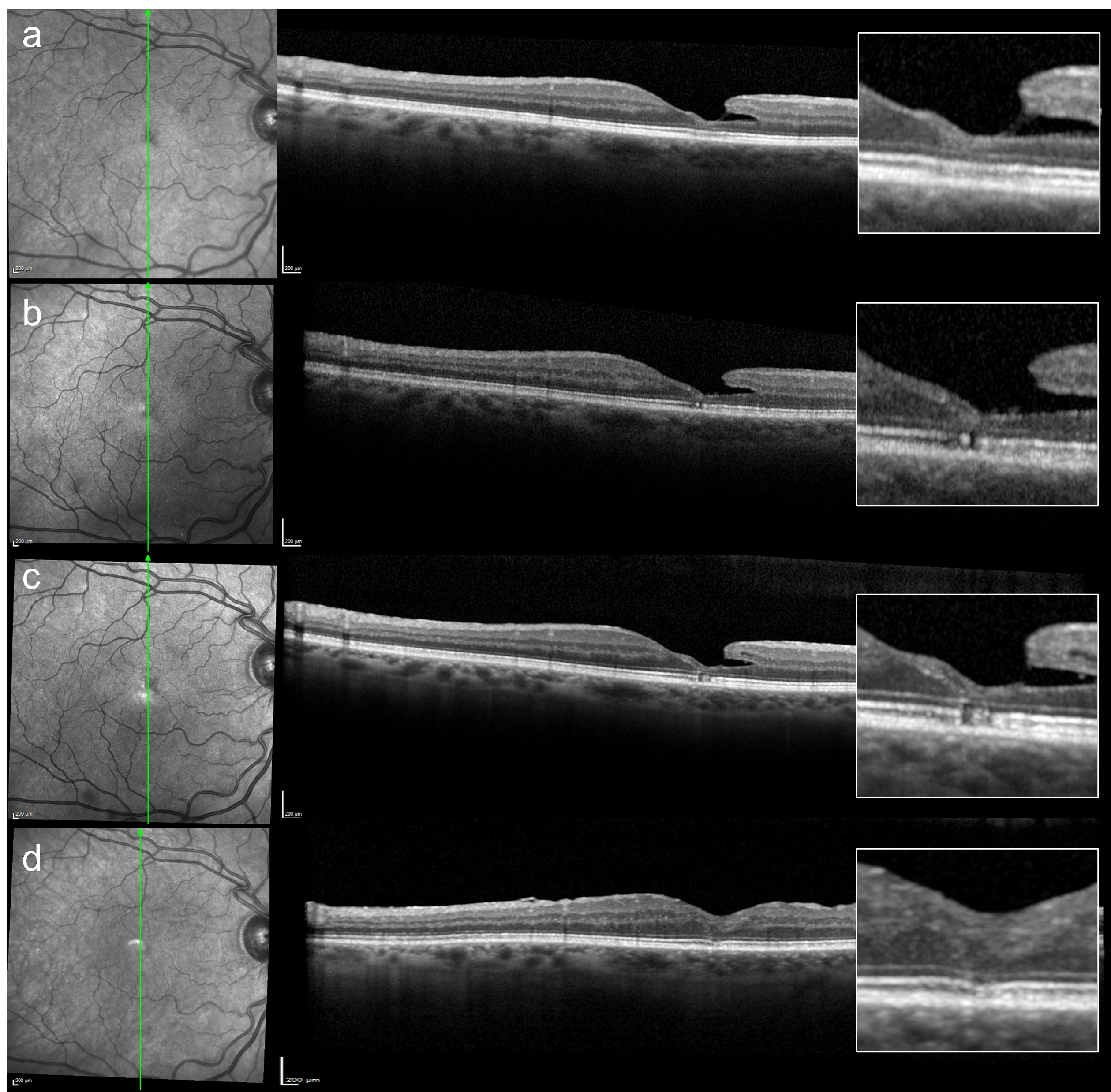

Supplement: Supplementary file 4 [file Image_1.TIF]

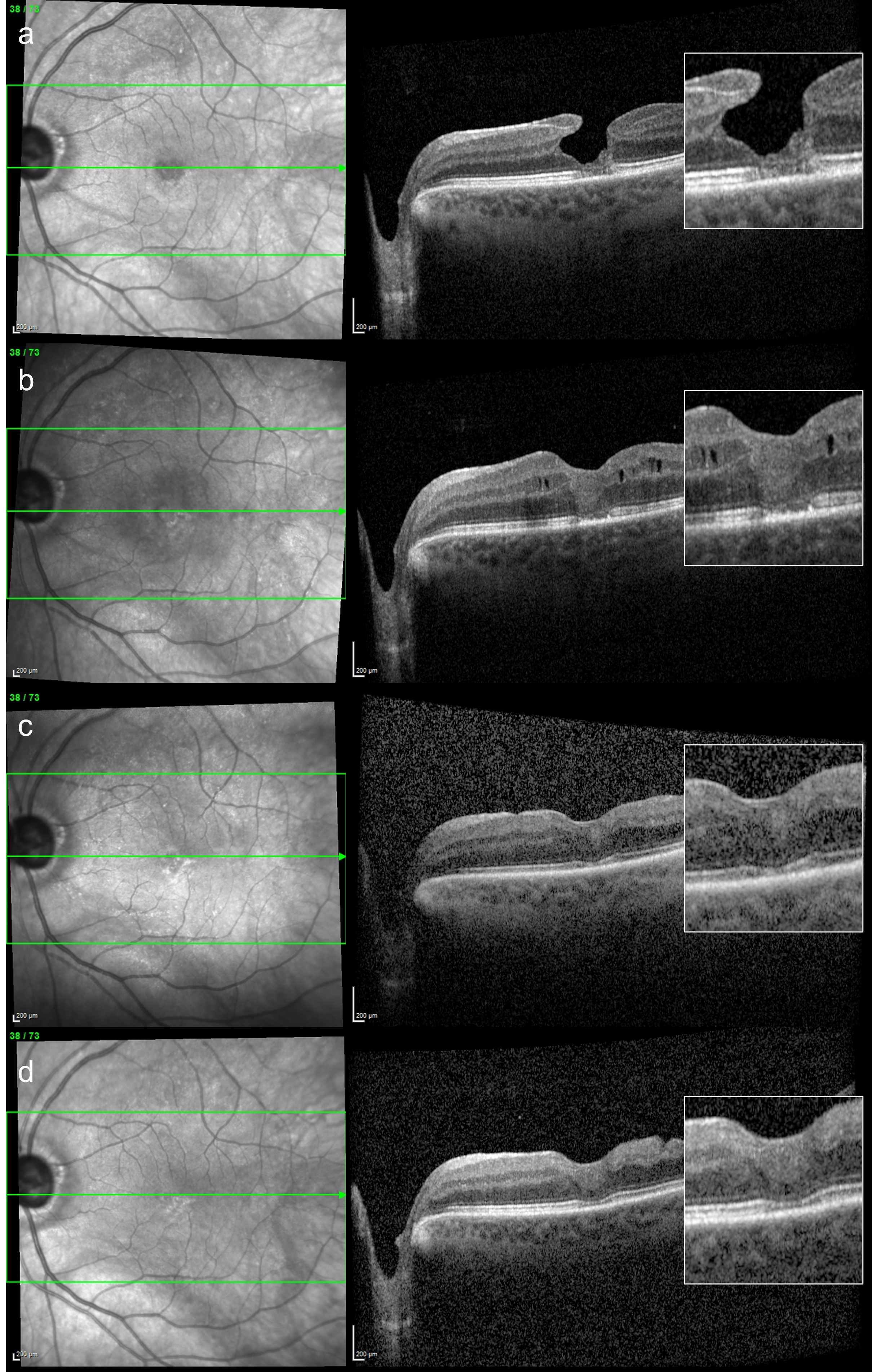

Supplement: Supplementary file 5 [file Image_2.TIF]

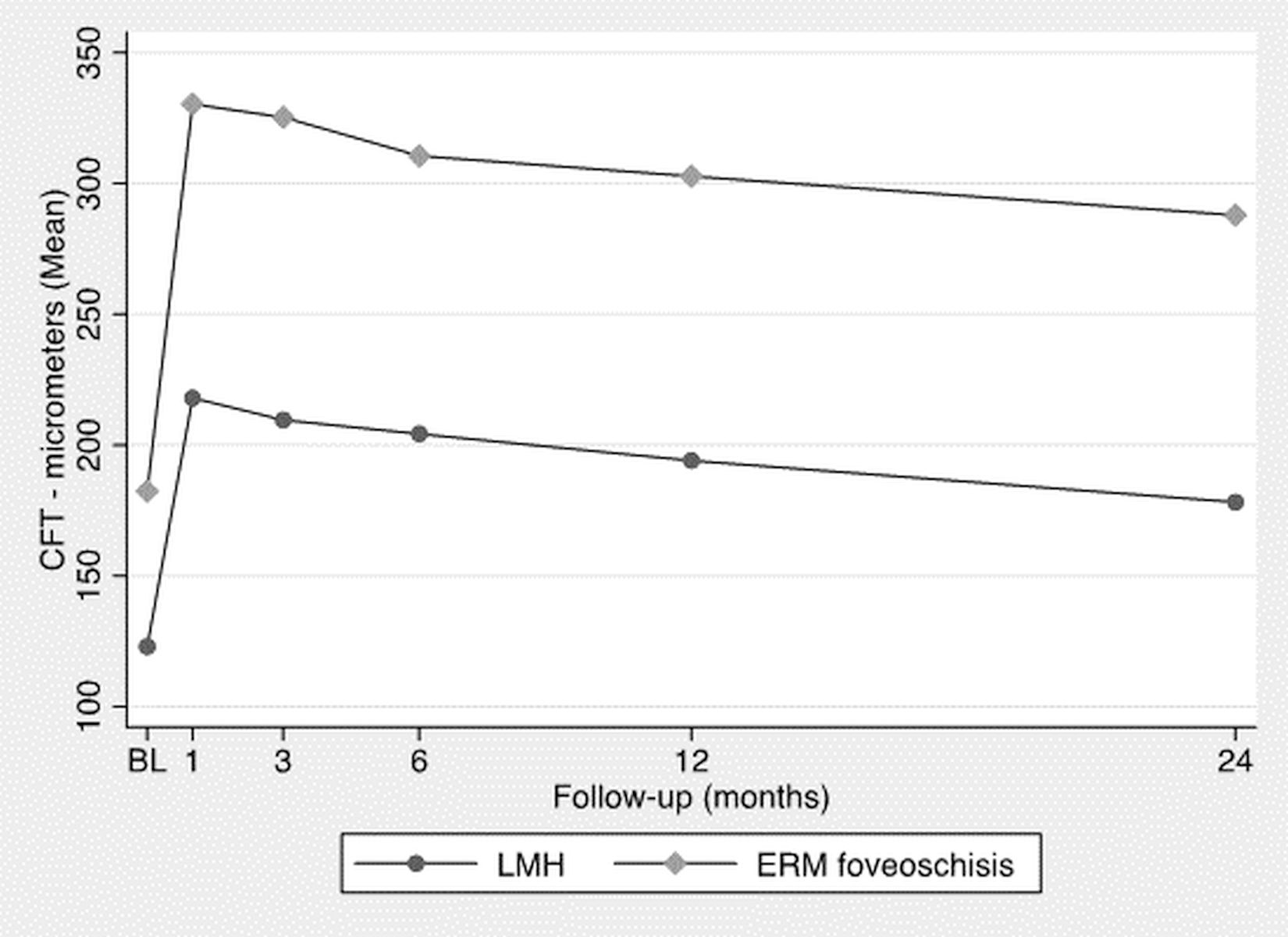

Supplement: Supplementary file 6 [file Image_3.TIF]

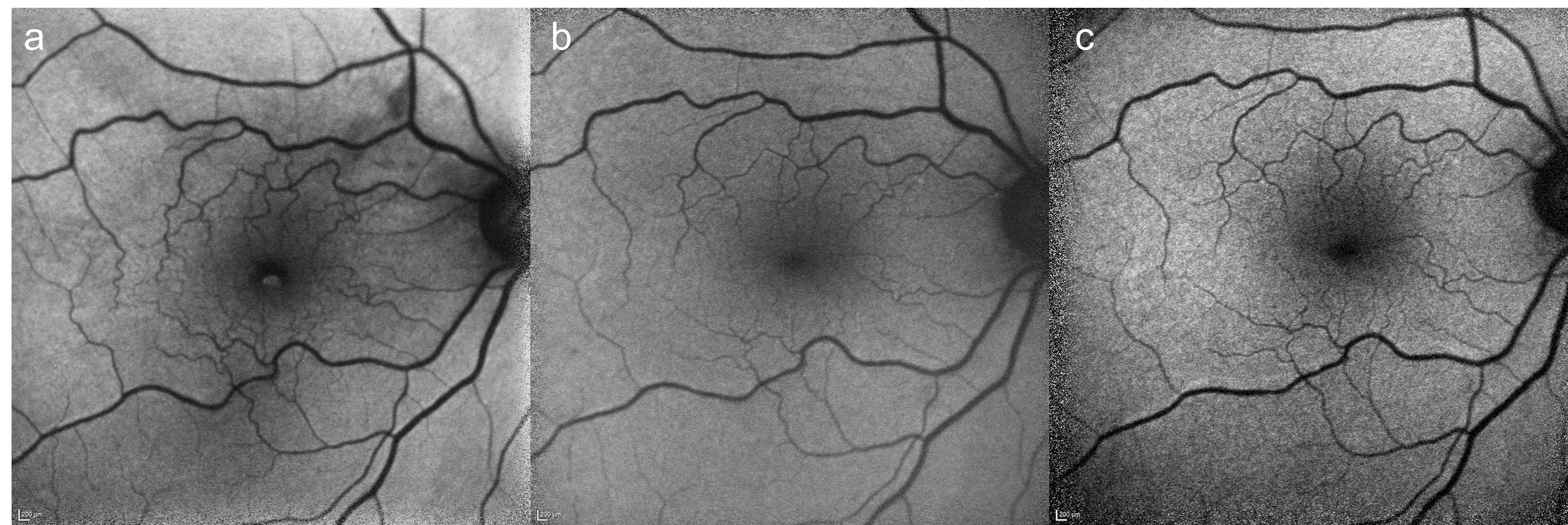

Supplement: Supplementary file 7 [file Image_4.TIF]
